# General practitioners’ management of occupational diseases: a qualitative study in French-speaking Switzerland

**DOI:** 10.1186/s12875-025-02888-w

**Published:** 2025-07-02

**Authors:** Gaubicher Coline, Staeger Philippe, Danuser Brigitta, Abderhalden-Zellweger Alessia, Krief Peggy, Mediouni Zakia

**Affiliations:** 1 Department of Occupational and Environmental Health, Center for Primary Care and Public Health (Unisanté), University of Lausanne, Lausanne, Switzerland; 2 Department of Ambulatory Care, Center for Primary Care and Public Health (Unisanté), University of Lausanne, Lausanne, Switzerland; 3 School of Health Sciences (HESAV), University of Applied Sciences and Arts Western Switzerland (HES-SO), Lausanne, Switzerland

**Keywords:** Occupational history, Work-related disease, Occupational disease, General practitioner, Occupational physician, Qualitative analysis, Prevention, Medical billing

## Abstract

**Background:**

Work-related health problems are a frequent reason for consultation with primary care physicians; however, few studies report on the way general practitioners’ (GPs) approach work-related health issues with their patients.

**Aim:**

To investigate the practices of Swiss GPs in regarding work-related health issues presented by their patients, and to identify the resources and difficulties they faced.

**Methods:**

Semi-structured interviews were conducted with 12 GPs, transcribed, and analyzed thematically.

**Results:**

GPs do explore their patients' occupational field. However, the data collected are limited, even though doctors recognize that a high proportion of mental and musculoskeletal disorders may be work-related. Work-related disease insurance issues are a frequent concern in GPs everyday practice, often causing a discomfort regarding these issues. Exchanges between GPs and employers regarding work-related health issues are relatively uncommon but can yield positive outcomes when they occur. Occupational risk prevention is confined to certain high-risk exposures. When asked to express their difficulties in dealing with work-related issues, GPs mention the lack of dedicated time, insurance issues, contact with employers or the lack of it and lack of training. As for resources, GPs mainly identify specialist doctors, but still, only few of them mention occupational physicians.

**Conclusions:**

Some work-related pathologies, whether frequent or serious, are not systematically investigated by GPs. This raises the question of their detection and probable under-reporting. To improve anamnesis, identification, and referral practices for occupational health issues, we suggest a targeted training for GPs, strengthened networking among stakeholders—such as occupational physicians, employers and insurers- and a revision of medical billing structures to better support the management of work-related diseases for both GPs and occupational physicians.

**Supplementary Information:**

The online version contains supplementary material available at 10.1186/s12875-025-02888-w.

## Background

Work-related complaints account for 15–25% of the reasons for consulting general practitioners in Switzerland [1, 2]. Given the small number of occupational physicians in Switzerland [2, 3], the management of these problems relies primarily on general internal medicine practitioners (GPs) and on certain specialists.

In Swiss occupational medicine, a distinction is made between occupational diseases (ODs) caused exclusively or predominantly by work, harmful substances or certain types of work [4]; work-aggravated diseases whose development is influenced by occupational exposure [5]; and work-related diseases whose cause cannot be attributed predominantly to work [6, 7].

The role of the GPs in occupational health is specified in the objectives of the Swiss medical curriculum [8], in the doctor's obligations to be accountable to his patient [9] and in the World Health Organization recommendations [10]: GPs should intervene in the identification of occupations and sectors at risk for workers'health and in the establishment of a link between pathologies and occupational exposure via occupational anamnesis, certify based on a medical examination a person's ability to work in a given job, support patients in declaring suspected occupational diseases and help patients design return-to-work strategies. GPs, like all physicians, report occupational diseases that require medical treatment, cause incapacity for work or result in death. They are also entrusted with the task to support patients and inform them of their social rights, such as compensation in the event of recognition of an occupational disease. So, GPs are the key player in the follow-up of patients who have been or are exposed to occupational hazards, given the long latency of certain work-related pathologies (e.g., cancers), which can appear long after workers have retired.

These tasks are complex. A French publication has shown that around half of GPs faced with a case of lung cancer fail to raise the question of asbestos exposure [11]. Yet, incomplete occupational history reduces the probability of including ODs in the differential diagnosis [12]. The existing literature also points to a lack of procedures with insurances and limited recourse to occupational physicians [13].

In cases of long-term health impairment accompanied by functional limitations affecting the patient's ability to work, it is essential for the general practitioner to actively support the key factors facilitating return to work. This includes developing a realistic strategy and timeline in collaboration with the patient and other relevant stakeholders. The objective is to enable patients to resume work as soon as their health condition is stable enough to minimize the risk of failure.

The Swiss security system briefly includes [14–16]:

- loss-of-earnings daily allowance insurance to compensate for loss of earnings due to ordinary illness (not compulsory, employer decides whether to take out this insurance or to pay for employees'absences (limited duration depending on seniority in the company, employees are advised to take out private insurance in this case));
- accident insurance (compulsory for employees), which covers the cost of benefits in kind (e.g. treatment) and in cash (e.g. daily allowances, invalidity or survivors'pensions) in the event of an occupational or non-occupational accident or occupational disease;
- invalidity insurance, the main player in professional reintegration and rehabilitation, is a key player in helping people return to work and remain in employment. Unlike accident insurance, it covers the deemed long-term consequences of any damage to health to maintain the autonomy of insured persons (e.g. reintegration measures, training, adaptation of workstations or outplacement).

For the latter two insurances, requests for notification of occupational disease and invalidity or early detection, respectively, are made by the insured person and by those involved (doctors, employers, family members, other insurances).

In Switzerland, companies are not obliged to systematically join an occupational health service (occupational physician, other occupational preventers) as in some other countries such as France, Belgium and Germany [17]. GPs can refer patients to occupational physicians, when there is one in the enterprise, which is rare. Employers mandate an occupational physician according to their interest in investing in occupational health, although there is a directive on"calling in an occupational physician or other specialists in occupational safety"which sets out the requirements [18].

An occupational physician can also intervene as part of a service mandate with the invalidity insurance system after it has been declared, when there is no occupational physician in the company [19].

To support general practitioners and their working patients, some university occupational health centers have set up occupational medicine consultations, providing direct access to an occupational physician, as it is the case in Lausanne and Zurich.

It should be noted that, for the time being, occupational medicine in Switzerland is the only medical specialty not to have TarMed points, a national pricing system used to calculate outpatient medical services in doctors'surgeries and hospitals and enabling doctors to be remunerated by the health insurance scheme [20].

The aim of this study is to investigate the practices of Swiss GPs in relation to the professional problems encountered by their patients, from anamnesis to therapeutic and insurance issues, and to characterize their resources and difficulties. Proposals will be made to support good practice.

## Methods

### Study design

A qualitative design was used to gain a comprehensive understanding of GPs’ practices in relation to occupational issues encountered by their patients [21, 22].

This study was conducted as part of an unfunded research project and the results were incorporated into a MD thesis at the University of Lausanne.

### Recruitment of participants

Participants were recruited through convenience sampling. The researchers'network facilitated access to the target population. GPs from the canton of Vaud were recruited from a database of 140 GPs who are internship supervisors for the Medical School of Lausanne, all of whom were eligible to take part to the study. The number of participants was set at 12 a priori, based on the experience of researchers having used qualitative methodologies. Sampling for the study was purposive. Recruitment considered an equal distribution between men and women on the one hand and between physicians practicing in urban and rural areas on the other. Potential participants were contacted by e-mail. Of the first 55 GPs contacted from the teaching GPs list, 18 agreed to take part in the study. Of these, 12 were selected to ensure a balanced distribution between men and women, and between doctors working in urban (≥ 14 0000 residents)/rural & peri-urban areas (< 14 000 residents). The validity of this a priori sample size was confirmed through data saturation at the end of the interviews. Despite the small sample size, it was deemed acceptable and sufficient to capture the diversity of GPs’ experiences [23].

### Data collection

A semi-structured interview grid (Appendix 1) was developed by the first author in consultation with 3 co-authors who are occupational physicians from the Department of Occupational and Environmental Health (DOEH) and tested with 2 GPs. Face-to-face interviews with participants at their offices, which lasted between 30 and 45 min, were conducted between September 2019 and January 2020. They were recorded, and transcribed verbatim.

### Data analysis

Data were analyzed thematically following Braun and Clarke [24, 25] recommended steps for reflexive thematic analysis. The first and last authors employed a deductive an inductive approach to independently code the participants'interviews. The researchers familiarized themselves with the transcripts, identified themes and sub-themes and classified all the quotations extracted from the transcripts according to the sub-themes list using the software MAXQDA. The classification of the extracts was discussed by these authors until a final classification consensus was reached. No specific software was employed for this purpose. Themes and subthemes were created and stored in an Excel file.

### Ethics

The study does not fall within the scope of the Swiss Human Research Act as it analyzes medical practices and does not use medical or personal patient data. Prior to the interviews, the participants were briefed on the study objectives, methods and guarantee of anonymity. Their consent to participate in the study and to record the interview were obtained in writing (return of the participation email) and orally (confirmation during the interview).

## Results

Twelve GPs were selected (Table 1). The mean age of the participants was 47.6 years. In average, they had obtained their Swiss diploma in internal medicine 18 years previously.

**Table 1:** Socio-demographic characteristics of the sample

Pseudonym	Age (years)	Sex	Years of GP experience^a^	Place of practice^b^	Country of postgraduate training
Dr A	53	Woman	19	Urban	Switzerland
Dr B	40	Man	15	Urban	Switzerland
Dr C	61	Woman	35	Urban	Switzerland
Dr D	42	Woman	6	Rural	Switzerland
Dr E	40	Man	14	Rural	Switzerland
Dr F	59	Man	26	Urban	Switzerland
Dr G	38	Woman	7	Urban	Switzerland
Dr H	49	Man	3	Rural	Switzerland
Dr I	46	Man	21	Rural	Switzerland
Dr J	47	Woman	22	Rural	Switzerland
Dr K	49	Woman	9	Urban	Switzerland
Dr L	47	Man	14	Urban	Switzerland

^a﻿^Years of experience since obtaining FMH (Foederatio Medicorum Helveticorum) GP diploma

^b﻿^Urban ≥ 14 0000 residents and rural/periurban < 14 000 residents

Six main themes were identified in the participants'discourse (Table 2): asking patients about work during the consultation, work-related illnesses, insurance aspects, contact with employers, prevention, difficulties in the management of work-related pathologies, and resources in the management of work-related pathologies. Of them, five themes were derived deductively as directly linked to the question within the interview grid, and one theme emerged inductively from the discourse of the participants.

**Table 2:** Themes and sub-themes

Themes	Sub-themes
Asking patients about work in the GP consultation	- Type of data collected - Data collection objectives - Use of a systematic method for data collection - Update of patients’ occupational records - The case of retired patients
Occupational diseases and work-related diseases reported by GPs	- Psychosocial risks - Musculoskeletal disorders - Occupational respiratory diseases - Occupational allergy and toxicology disorders - Occupational cancers
Insurance-related issues	- Work incapacity certificates - Occupational disease detection and reporting - Disability insurance claims - Relations with insurance doctors
Contact with employers	- Benefits - Difficulties
Prevention	- Primary prevention - Secondary prevention
Resources for managing work-related pathologies and their limits	- Referral to occupational health specialists - Referral to other medical specialists - Training options - Complexity of the insurance system - Lack of knowledge of the patients working conditions - Lack of training - Lack of consultation time - Overall deterioration of working conditions
Emerging theme	
Return-to-work	- Accompanying patients return-to-work - The interaction with employers in this context, negotiation and adaptation

### Asking patients about work during the GP consultation

The findings of this study indicate that most of the study participants described adopting a global perspective when addressing health issues, including questions pertaining to occupational and socioeconomic factors. The occupational situation may be addressed as the main reason for consultation, or because a health problem has repercussions on the ability to work.

*"That's when we talk about their professional activity [when they have to stop working]" *(Dr. C)

### Occupational and work-related diseases reported by GPs

#### Psychosocial risks

Participants were almost unanimous in declaring that psychosocial risks (PSRs) are at the forefront of work-related health problems particularly the “burnout” syndrome. Other common pathological situations included work-related stress, depression, anxiety disorders, adjustment disorders, mood disorders or persistent fatigue. Some occupational groups were identified by GPs as at risk of facing specific issues. For instance, farmers were identified as a professional category with a high risk of suicide.

Facing PSRs, some participants use active listening, give personal development advice and coaching, in addition to prescribing sick leave certificates. Participants sometimes find themselves at a loss, and hand over to the specialists. Some of them emphasize the importance of collaboration with psychologists and psychiatrists when it comes to health insurance aspects.

*"Over the years, we've developed all kinds of strategies! First of all, you can look at what patients have already done... I think listening is still a good way to de-stress" *(Dr. J)

#### Musculoskeletal disorders

The musculoskeletal disorders (MSDs) frequently encountered by participants are low back pain, tendonitis, epicondylitis, scapulalgia and nuchalgia. Problematic biomechanical exposures such as forceful work and repetitive movements are identified. The need to understand the workstation is highlighted. Referral to specialists is mentioned without including occupational health specialists*.* Chronic MSDs in patients at the end of their careers are identified as a particularly difficult situations to manage, because of the repercussions on employability, with a high risk of forced professional retraining.

*"[…] Then, in the older patient population, there are all the osteoarticular problems that lead to work incapacity, and that's where it gets very, very, very complicated, in saleswomen, cleaning ladies, janitors." *(Dr. F)

#### Occupational respiratory diseases

Occupational respiratory diseases carry less weight than psychosocial risks and musculoskeletal disorders in the participants'discourse. The conditions mentioned by the participants, but not necessarily encountered, are asthma, silicosis, pulmonary fibrosis, and asbestos-related cancers. For treatment, referral to a specialist seems to be the rule.

#### Occupational allergy and toxicology

Allergies and occupational intoxications were not commonly mentioned. The allergens cited are perfumes, resins, and flour, with respiratory and dermatological consequences: asthma, contact dermatitis and hypersensitivity syndromes.

Clinical entities can be imprecisely described and mistaken for other diseases:

*"The example was a fairly young patient, I think 25, a baker, […] who had gluten intolerance, which is therefore an allergy, and through the allergology consultation, this was highlighted […]."* (Dr H)

When it comes to treatment, referral to specialists again seems to be the preferred option.

#### Occupational cancers

Apart from asbestos-related cancers, little is mentioned about occupational cancers by the participants. No participants had diagnosed occupational cancer in their past practice. Some even question their knowledge of the subject:

*"I'm wondering about cancers, it's often ear nose and throat cancers, carpenters [...]. I don't have it in my head: sometimes we forget."*(Dr. J)

### Insurance-related issues

Participants note the omnipresence of the certificate of incapacity for work (Swiss sick leave certificate) in their practice. The complexity of the exercise is widely commented upon. Participants explain that determining the duration of work incapacity tends to be difficult, and that reassessment is a valuable tool*.* Some feel that the responsibility involved with making these certificates is too great. Dealing with uncertainty seems difficult:

*"[...] it's a kind of false certificate for me, because my certificate should be purely medical, which it never is!" *(Dr. I)

As for using a systematic method for establishing incapacity for work, all the participant GPs answer negatively, although a distinction is made according to the pathology: circumscribed or not, somatic or psychic in origin.

Participants explain that they rarely notify work-related diseases to accident insurers (Swiss health insurers for occupational diseases). They explain this by the lack of opportunity to do so, by the uncertainty surrounding the conditions of this declaration or by their ignorance of the procedure:

*"Yes, caused in part, but I don't know how it's recognized by the insurances, sometimes it's a doubt." *(Dr. C)

On the other hand, they declare that they frequently have to must deal with loss-of-earnings insurers. Participants tend to describe difficult relationships, with the impression that insurers'decisions are sometimes hasty. The imperative nature of the insurances requests is emphasized by certain participants; distinction is made between serious somatic illnesses, for which insurers rarely question certificates, and low back pain or psychological disorders, for which detailed arguments are generally demanded. To some GPs, challenges to the medical officer's decisions are a matter of"negotiation"more than “collaboration”, that leads some participants to question the professional ethics of their interlocutors. In other cases, insurance companies’ interventions are seen as positive, for instance when an incapacity for work would otherwise be prolonged inappropriately. 

### Prevention

Primary prevention of occupational risks is sometimes addressed during office visits, but most participants focus on secondary prevention. Apart from preventive advice provided by accredited doctors for specific working environments (e.g. extreme cold weather) is the pre-apprenticeship visit is cited by some participants. It is described as a privileged moment for primary prevention where GPs are able to identify health situations incompatible with certain professions and to give young people advice on maintaining good health in the long run (diet, physical activity).

Some conditions, such as MSDs and PSRs, also offer opportunities for secondary prevention advice. In the case of MSDs, workstation adjustments and avoidance of other triggering factors are suggested. Regarding psychosocial risks, respondents describe various strategies, mainly active listening, and stress management advice. However, the participating GPs recognize the limitations of their action, prevention wise:

"*[…] for the heavy physical work, it's complicated […] there we are quite reactionary and not so much preventive.*" (Dr. G)

### Resources for managing work-related disease and their limits

Medical specialists are identified by the participants as a resource for confirming a diagnosis, issuing long-term incapacity certificates, or facilitating communication with employers when direct contact proves challenging. Specialists are also called upon to respond to insurers'requests. No mention is made of occupational physicians during the interviews. The participants are familiar with the DOEH (Department of Occupational and Environmental Health, a university public service within Unisanté, Lausanne that aims to promote healthy working environments and conditions that are favorable to individuals and society) and all of them have contacted this service at least once in their career. However, their knowledge of its services is limited*.*

However, there are some difficulties and lack of resources, also reported by the participants who globally consider that occupational anamnesis, and management of work-related health problems are too time-consuming:

*"It would take an hour to see the impact on health and an hour to see what their job is, what we can do […]*" (Dr. C)

The complexity of the social insurance system, including that for occupational diseases, and the lack of knowledge of the procedures for declaring occupational diseases, are also mentioned. Another difficulty is the lack of objective assessment of working conditions, due to a lack of contact with employers. The lack of training in occupational health and safety is also mentioned. Some participants report limited skills in assessing functional limitations, which are necessary for determining residual work capacity.

*"I find that given the large mass of work-related problems we encounter, training isn't sufficient, so it should be improved, because it's true that we're very often dealing with occupational health problems." *(Dr B)

More generally, participants tend to deplore the deterioration in their patients'working conditions and the increasing demands for productivity in the world of work.

### Return to work

Returning to work lies at the crossroads of complex medical, legal, economic, and social concepts, and involves a multitude of stakeholders who are often disconnected from the realities of medical practice. In daily practice, while the patient should be the main active player in the return to work process, he often sees the GP as the person to talk to in the event of sick leave, it can be difficult for him or her to grasp all the determinants of the return to work (e.g. the reality of the workplace). Some participants describe situations where their help is required:

*“Often we have tried to find arrangements with people, that is to say, can we find an arrangement with the employer so that this is possible or can we encourage the person to retrain or find other solutions.” *(Dre C)

Several GPs describe individual cases where they had to help patients along to get back to work:

*“I had a female patient who worked in the kitchen and who had eczema on her hands and it was related to the soap used, related to the product used and she also did cleaning in an requirement home; it was cooking and cleaning, she had to be reclassified, in fact, she did some training again, she is now a secretary in a company” *(Dre A)

According to the participants, communication between GPs and employers can sometimes lead to good results when it comes to return to work, or even if people stay at work part-time in the company. Collaboration between employers and GPS can involve adapting the percentage of work incapacity, adapting tasks, or helping the patient to return to work after prolonged sick leave. In the last case, therapeutic resumption or professional retraining have been described. However, the participating GPs do not often contact employers, mostly because they think that patients are generally reluctant to share some pieces of information with their employers, agreeing with the patients on the point that the process is complex or even risky for them. The participants tend to think that it is difficult to talk to employers while protecting medical confidentiality.

*"[...] very positive situations! But a lot of people have to get involved! There has to be a company policy along these lines [...]" *(Dr. D)

## Discussion

This study provides insights into the way GPs approach and manage work-related problems in the Canton of Vaud. This is a first attempt to analyze the practices of GPs in relation to work-related issues in Switzerland. Using a qualitative methodology, we were able to identify some major trends in current practices.

In GPs practice, gathering information about patients'working environments usually happens during the initial anamnesis or at the occasion of a work-related complaint. It is more reactive than proactive. As shown by some studies, it is often limited to the job title [26].

The most frequent ODs and work-related diseases that are cited by the participants in this study correspond to what has been described in the literature [1]. The same is true for work-related diseases, notably psychological illnesses. However, GPs mention limited experience of ODs reporting and tend to leave this task to specialists without really checking whether it has been done. Indeed, a Swiss study reported that 38% of GPs questioned out of a panel of 453 had never declared an OD. Only 7% reported knowing how to declare one. Among those who had already established an OD declaration, only 9% had done so more than 10 times in their careers [1]. An Italian study found that only 9% of the GPs surveyed had ever made at least one occupational disease declaration [27]. Yet, the proportion of occupational etiologies is significant in many diseases: occupational asthma accounts for 15% to 20% of new cases diagnosed in adults and to 2 to 8% of cancers [28, 29].

The under-reporting of occupational diseases may be due to a lack of occupational health training for GPs, of information on the patients’ workstations, of knowledge on how to report occupational diseases to accident insurers, and to the lack of time to do so [27, 29–31]. Certain multifactorial diseases, such as cancers, have a long latency period, which makes it difficult to establish a link with the profession. In Switzerland, around 2,000 new cases of cancer could be recognized as occupational diseases each year, compared with only 150 currently recognized by accident insurers [32, 33]. The under-reporting of occupational disease results in reduced social protection for the patient and/or his or her dependents, makes the health impact of occupational exposure invisible and limits the scope for collective preventive action in companies [28].

Other insurance procedures, particularly the drafting of work incapacity certificates, are very much a part of GPs'day-to-day work, with up to a quarter of medical consultations resulting in the issue of such a certificate [34]. The task is seen as complex, not only in this study but also in others, not only from the point of view of the technical details involved in determining the work capacity, but also because of the ambivalent position of the GP as a caregiver who must enable the patient to maintain a good state of physical and mental health, and as an expert, who has a responsibility to judge work capacity for the insurances [35].

Another finding of our study is that prevention of occupational diseases is limited and unsystematic in GPs’ practice, in line with a Swiss study that showed the preponderance of other preventive health topics such as physical activity and addictions [36]. Moreover, the official table of prevention recommendations published by EVIPREV (Evidence based preventive medicine) does not include occupational risks factors [37].

GPs resources appear to be limited. The existing ones are under-exploited. A 2012 Swiss quantitative study [1] reported that 47% of GPs refer the patient to an occupational physician when the patients’ work is identified as the main cause, but it remains rare for most of them (64%). However, of those who do, 94% consider it useful. The reasons given by those who don't are that they don’t know an occupational physician personally, prefer to ask other specialists, don’t consider it necessary or never thought of it. Only 1/3 of the GPs surveyed said they were familiar with, or had already used, the Department of Occupational and Environmental Health at Unisanté in Lausanne [1]. The low recourse to occupational medicine could be explained by the low number of occupational physicians in Switzerland (130) and by the relatively recent recognition of the specialty of occupational medicine by the Swiss center for postgraduate and continuing medical education in 2001 [2]. The absence of specific pricing for occupational medicine or TarMed points in the price list for outpatient medical services in Switzerland- may also contribute to the situation. This would also explain why the majority of occupational physicians in Switzerland work directly with companies. Once these aspects of valuing this collaboration have been settled to allow equitable access to preventive care, it would be relevant in Switzerland to investigate the criteria for referral to the occupational health physician by the general practitioner [38, 39], the image and perceptions of occupational health physicians by general practitioners and vice versa in order to observe certain barriers to multidisciplinary collaboration [40–42] and work to improve it [43] as has been done in Germany. GPs’ collaboration with other specialists (psychiatrists, pneumologists, allergologues etc.) is present, in line with a study specifically centered on the management of chronic depression in French-speaking Switzerland [44].

Among GPs difficulties, the lack of training in occupational medicine is central. It is deemed insufficient by participants, although it is complicated to assess where the shortcomings lie. Requests are often related to insurance procedure knowledge, identification of occupational risk factors, and learning about effective prevention tools. Indeed, occupational medicine training in Switzerland is limited in medical pre-graduation studies. The average number of hours is less than 10, whereas the European median is 33 h [45]. Some faculties do not offer occupational medicine courses. Post-graduate training in occupational medicine for GPs is lacking because graduation objectives do not include occupational health, and no training period in occupational medicine is required to get the diploma [2]. In a study including 254 doctors in Germany [46] 91% of doctors declared they had underestimated the importance of occupational medicine knowledge during their pre- and post-graduate training. Once in practice, 75% of these practitioners had not received any further training on the subject, although 25% considered their knowledge to be insufficient. Finally, academic resources are limited. In Switzerland, there is currently only one professorship for occupational medicine (Zürich). The potential for research in occupational medicine is thus limited [2].

The lack of time described by GPs for dealing with work-related issues is recurrent, although hardly surprising given the shortage of GPs in Switzerland and the TarMed service-based pricing system.

Indeed, the system makes no provision for points dedicated to occupational history-taking, contact with employers or the organization of return-to-work networks with employers and insurances. This work is not valued, which is inconsistent with the Profiles objectives of the current medical curriculum [2].

This study has some limitations. A bias in participants selection is possible because the role of student supervisor may select a particular profile of GPs. The study is confined to the Canton of Vaud, but this limitation can be tempered because most conclusions seem at least partly can be transposed to Switzerland as a whole (under-reporting of ODs, too few occupational physician posts, limited pre-graduate training provision). Finally, this research is limited to GPs point of view obscuring the multidisciplinary nature of occupational health issues.

## Suggested actions to support GPs in addressing occupational health issues

To support good GPs practices, action can be considered at different levels of intervention. This is complicated because each level involves different stakeholders (doctors, employers, insurers, public policies), who need to collaborate to co-construct operational practices. Here, we propose some ideas that could drive future action and research.

### Strengthening training in occupational medicine for GPs

It would be useful to establish consistent training objectives for pre-grad and post-grad students, and for GPs in continuous training, to make occupational medicine courses accessible to all students/GPs and increase the number of hours dedicated to this teaching. Emphasis could be placed on occupational anamnesis, major occupational diseases and risk factors, assessment of functional capacities and completion of sick leave certificates. With that in mind the National College of Occupational Medicine Teachers has drawn up an occupational medicine referential for medical students and primary care physicians [47].

Increase GPs'awareness of factors that promote or impede return to work, and promote exchanges with employers, could be an asset for GPs in the management of certain complex work incapacity situations. Some tools already exist for the assessment of patients'functional limitations, such as the WOCADO, a method for assessing work capacity with a view to returning to work, or the Resource-Oriented Integration Profile (PIR) [48], developed by Swiss Insurance Medicine. However, these tools are rarely used. They could be more actively promoted among GPs and made more operational, to support and standardize stages of returning to work and to facilitate information exchanges between stakeholders (insurance companies, employers).

### Promoting integrated patient care among stakeholders

Integrated, holistic patient centered care is an important issue in general medicine and occupational medicine, starting with the benefits of closer collaboration between GPs and occupational medicine specialists. Occupational medicine contribution should be made more visible to GPs: contact with the employer, assessment at the workstation, support for returning to work, diagnosis assistance in case of suspected occupational disease, help in carrying out occupational disease reporting procedure, etc.). More frequent exchanges between GPs and employers should be carried out to refine incapacity certificates and the conditions for returning to work after a period of incapacity. Better communication between GPs and insurance companies should be encouraged, as well as greater transparency in insurers'decisions regarding refusal of services, to avoid costly and time-consuming expertise and enhance mutual trust between stakeholders.

### Improve billing for the detection of work-related illnesses and the coordination of return to work

Regarding the pricing of work-related services, there could be a strong impact from integrating specific tariffs dedicated to occupational risk prevention for both GPs and occupational physicians, covered by compulsory health insurances. This would enable a better occupational anamnesis and therefore a better detection of ODs and would make it possible to support the return to work and job retention.

## Conclusion

Our study shows that GPs can sometimes find themselves in an uncomfortable position when it comes to occupational health, in all its dimensions. Further studies are needed to clarify certain trends highlighted by this study and to identify concrete areas for improvement, such as collaboration with specialist doctors (including occupational physicians), employers, insurance companies and patients in a holistic, network-centered approach to the patient. Meanwhile, to improve their practices in terms of anamnesis, identification, and referral of patients with work-related health problems, we propose avenues such as training for GPs, improved networking, and recognition of related services—currently rare—by the billing system.

## Supplementary Information

Supplementary Material 1.

## Data Availability

The transcribed texts of the interviews are available on request from the authors in the Unisanté datahub.
