# Supplementary material for: General practitioners’ management of occupational diseases: a qualitative study in French-speaking Switzerland
Source: BMC Prim Care. 2025 Jul 2;26:207. doi: 10.1186/s12875-025-02888-w (PMC12220256; doi:10.1186/s12875-025-02888-w)
Supplement: Supplementary file 1 — Supplementary Material 1. [file 12875_2025_2888_MOESM1_ESM.pdf]

Supplementary material: Semi-structured interview guide used during the study participants interviews.

“I'm currently in my 4ème year of medicine at the University of Lausanne and I'm doing a thesis, in conjunction with Unisanté's Department of Occupational and Environmental Health (DOEH), formerly known as the Institut Universitaire Romand de Santé au Travail. The aim of my research is to investigate how patients' work-related issues are addressed by general practitioners.

This interview should last between 30 and 45 minutes. What's interesting in my research is to understand your real, concrete practice when faced with medical situations that include professional aspects.

As agreed, we guarantee complete anonymity.

1)

The interview will be recorded, transcribed and analyzed. Do you agree?

2)

Generally speaking, how do you approach the subject of working with your patients?

<u>If you don't spontaneously ask about this, ask them to explain:</u>

What information do you ask the patient for? Which of these do you ask for systematically *vs.* occasionally?

Do you update this information in any way?

If the patient is retired, do you ask about their past professional life?

For what purposes do you collect this data?

If the person answers "No, I don't want to talk about it,

What specific clinical situations prompt you to tackle the subject of work?

3)

What do you consider to be the main occupational illnesses to watch out for?

4)

What is your approach to establishing a link between the patient's illness and his or her work in practice?

Can you think of a situation in which you have diagnosed or strongly suspected an occupational disease?

Can you tell us how it happened? *(If necessary, guide us by suggesting the following order: medical and professional history, establishing the link, declaration, contact with the company).*

Do you think you're well equipped, with your experience and knowledge, to detect clinical situations that could be cases of occupational disease?

What criteria do you use to decide whether to report a suspected occupational disease to the relevant authorities?

5)

When you identify a risky occupational exposure based on the patient's description of his or her work activities, do you adopt a preventive approach in certain situations?

If so, in what situations and how?

If not, how do you react when a patient comes to your consultation reporting intense stress at work, for example?

6)

What precautionary steps do you take?

Support for the declaration of occupational disease to LAA/Suva, work stoppage while LAA/Suva insurance makes its decision, etc.

7)

Finally, what difficulties do you encounter when dealing with clinical situations that may be related to your patients' work, and what resources do you have to help you? *(If necessary, guide the participant by proposing categories of factors: difficulties: lack of time, occupational health skills, training, interest, lack of transparency regarding recognition as OD by LAA/Suva insurance companies, etc.; resources: referral* to an occupational physician (from the patient's company or DOEH-Unisanté, ex-IST), referral to an organ specialist, etc.).

What would be useful to you (what are your needs)? (Training in occupational health, knowing which occupational physicians to refer to (list), support from a social worker for the patient's declaration of OD, etc.).

Thank you very much. To conclude the interview, here are a few additional questions about your situation:

1) Age
2) How long have you been practicing?
3) What type of structure do you work in? Independent, shared practice, hospital ?
4) Do you work in urban or rural areas?
5) In which country did you complete your post-graduate training for the diploma in internal medicine?
6) Do you have any qualifications other than the diploma in internal medicine?

The interview is over. Thank you very much for your time. Are there any important points we haven't covered, or simply things you'd like to add? Do you have any other questions? »
